# Why do older people with multi-morbidity experience unplanned hospital admissions from the community: a root cause analysis

**DOI:** 10.1186/s12913-015-1170-z

**Published:** 2015-11-27

**Authors:** Richard L. Reed, Linda Isherwood, David Ben-Tovim

**Affiliations:** Discipline of General Practice, Flinders University, GPO Box 2100, Adelaide, 5001 South Australia; National Institute of Labour Studies, Flinders University, GPO Box 2100, Adelaide, 5001 South Australia

**Keywords:** Hospitalization, Readmission, Root cause analysis, Elderly, Preventable hospitalization, Clinical error, Delay in care, Australia, Minimal care

## Abstract

**Background:**

Increasing demand for hospital services by older people is a major concern for Australian health care providers. To date there has been little in-depth research that encompasses contextual and systems factors contributing to hospital admissions. The objective of this study was to determine the reasons why older patients experienced unplanned hospital admissions to a major public hospital.

**Methods:**

A retrospective qualitative study using a Root Cause Analysis (RCA) methodology was conducted in a major public hospital in Adelaide, South Australia and surrounding community. Community dwelling older people admitted to the hospital who were well enough to give informed consent and be interviewed were invited to take part in the study. With patients consent, family members, general practitioners (GPs) and specialists were also interviewed and patient hospital records reviewed. Using a purposive sampling technique to obtain maximum variability, thirty-six older people (aged 70 years and older) participated in the study. GPs (*n* = 17), family members (*n* = 14), and other healthcare providers (*n* = 12) involved in their care were also interviewed. Cases were then analysed according to a standardized protocol to determine the root cause of admission. Root causes were then assigned to broader categories using thematic analysis.

**Results:**

The root causes of unplanned admissions were identified and categorised into six causal groups: a consequence of minimal care, progression of disease, home care accessibility, high complexity, clinical error, and delayed care-seeking by the patient.

**Conclusions:**

RCA can be effectively applied to determine the causes of unplanned hospital admissions although the process is time consuming. Four categories of admission (minimal care, clinical error, home care access, delayed care-seeking) were deemed potentially preventable. This methodology and classification approach may assist in designing interventions to prevent future hospitalisations in this high-risk population.

**Electronic supplementary material:**

The online version of this article (doi:10.1186/s12913-015-1170-z) contains supplementary material, which is available to authorized users.

## Background

Increasing demand for hospital services is a major factor in the growing cost for providing health care in Australia. Public hospital use has been steadily increasing at a rate of 3.2 % per year between 2006–07 and 2010–1 [1]. Older people are disproportionately high users of medical services, accounting for 38 % of all hospital admissions, and 48 % of total hospital days [1]. This population also accounts for the majority of the annual increase in hospitalizations [2].

Research on the causes of unplanned hospital admissions has predominantly focused on the prevalence of adverse drug events [3–5], and the identification of risk factors relating to emergency department presentations, hospitalisations, and early readmission [6–12]. A systematic review of the determinants of emergency department visits by older patients, found that perceived and actual poor health status, previous hospital/emergency department utilisation, and lack of access to primary health care services were the main risk factors for presentation to hospital [13]. Within primary health care, low continuity of care [8] and a lack of timely access to services [14, 15] have also been identified as increasing the risk of emergency department utilisation by older adults.

These risk factors for admission have generally been limited to data readily available in hospital records. They are likely to be mediated by important contextual and health system-related issues which are likely to differ between health systems. These mediating factors, however, may play an important role in the rising rates of hospital use. To date there has been very limited research that addresses these important contextual factors in an Australia. Using an in-depth approach, this study sought to determine the reasons why older patients with multiple health conditions experienced a unplanned hospital admission.

Root Cause Analysis (RCA), “a systematic process whereby the factors which contributed to an incident are identified” [16], provided the methodology for the study. Used routinely within healthcare services since the mid-1990s, RCA has traditionally provided a framework in the identification of the root cause(s) of serious adverse events. A novel application of RCA was applied to this setting, and was considered an appropriate methodology to better understand the reasons for unplanned hospital admissions.

## Methods

This study was undertaken in a tertiary public hospital serving a health region of approximately 400,000 people in the metropolitan Adelaide area. In-depth qualitative interviews were conducted with inpatients and their family members, GPs and other healthcare providers involved in their care. In order to be eligible for participation in the study, patients were required to be: (i) aged 70 years or older, (ii) living in the community, (iii) able to give informed consent for participation, and (iv) well enough to participate in an interview. Patients who were eligible for the study were identified upon admission to the acute medical unit through a review of the hospital ward notes and/or discussions with the nursing shift co-ordinator.

A purposive sampling strategy [17] was used to identify potential patients. If several patients were eligible to take part in the study on a given day, then cases which were likely to provide more information-rich data (e.g. admissions due to a pre-existing illness, previous inpatient admissions, involvement with primary health care or outpatient services) were selected where possible. Maximal variation was also sought with regard to gender and illness type.

Written informed consent was given by each subject in the study (the consent form provided an agreement to participate in an interview, access to medical records, and for the patient’s carer, GP and healthcare provider to be contacted for further information). Prior to the conduct of interviews with individuals involved in the patients’ care, an explanation of the study was provided and verbal consent obtained. The study was approved by the Southern Adelaide Clinical Human Research Ethics Committee.

RCA was performed using a series of prescribed stages. As this project was a new application of the methodology, the RCA protocols used by the SA Department of Health [18] were adapted to make them relevant for the study and are outlined below.

### Stage 1: Initial data collection (patient interviews, examination of medical records)

Face-to-face semi-structured interviews were conducted with patients during their hospital admission. An interview guide is shown in Additional file 1. The primary purpose of this interview was to establish a chronology of how the current episode of illness had developed and whether any previous health service interventions had occurred. Additional information was also collated regarding previous health concerns, admissions and treatments; contact with GPs, specialists and other healthcare providers; and the level and type of support received in the home.

### Stage 2: Event flow diagram constructed

An event flow diagram is a chronological diagram of the series of events leading up to an adverse event. In this study, the event flow diagram provided a visual tool to understand how the particular episode of illness developed and led to the admission to hospital. At each step of the diagram, questions were identified to help the RCA team better understand why each event had occurred. The team also identified who needed to be interviewed in order to obtain this information.

### Stage 3: Further data collection (interviews with family, GP, hospital and community staff)

RCA requires rich and detailed data in order for accurate ‘root causes’ to be identified. Therefore interviews were also conducted with family members, GPs, specialist physicians, and outpatient and community healthcare providers who were involved in the care of the patient. These interviews were conducted either face-to-face or over the telephone depending on the preference of the interviewee and were guided by the information obtained in Stage 1. An example is listed in Additional file 2.

### Stage 4: Cause and effect diagram constructed

The cause and effect diagram enables the identification of chains of causal links which lead from the root cause(s) and contributing factors to the specific event of hospital admission. Using the admission to hospital as a starting point, the question ‘why’ was asked repeatedly until the root cause of the admission was identified. Factors which may have contributed to this root cause were also noted.

An example of the final analysis for one RCA is listed in Additional file 3.

### Stage 5: Root cause attribution

A final stage of the analysis used thematic analysis [19] to identify overarching issues and themes arising from the individual patient RCAs. The thematic analysis was conducted through a comparison of the root cause statements and recommendations generated for each individual case in order to identify patterns within the data. Initial thematic groupings were first agreed by the investigators after a preliminary review of the cases. The admissions were then reviewed and root causes allocated under six causal categories. Where multiple issues were identified as contributing to hospitalization, the case was classified according to the predominant root cause. In the event of differences between investigators, a final disposition was reached by consensus.

## Results

### Study Sample

**Fig. 1:**
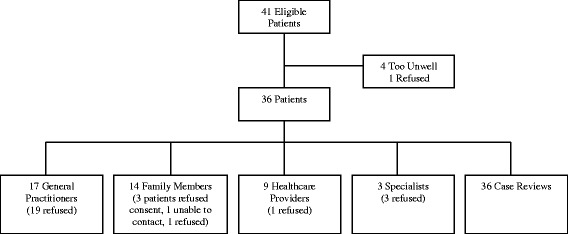
Study recruitment and participation

The recruitment process, and subsequent sample, for the study is outlined in Fig. 1. In total, 36 patients, 17 GPs, 14 family members, and 12 other healthcare providers participated in the study. Medical records were also examined for all of the participating patients.

The majority of the patient sample was female (68 %), reflecting both the predominance of older women on the hospital ward and their greater willingness to consent to participation. The ages of the patients ranged between 71 and 91 years (with a mean age of 81 years). Over two-thirds (78 %) of patients were from the metropolitan Adelaide area, with a further 22 percent living in rural locations. All patients were in independent living accommodation, with the majority (67 %) living in privately owned properties, 19 percent in retirement villages, and 14 percent in public housing. Just over half of patients (54 %) lived alone in their home, and 43 % lived with a spouse.

Support in the home was received by a majority (72 %) of patients. Of these, 46 percent received assistance solely from informal networks, while 54 percent received community care services. Formal support included cleaning services, meal provision, assistance with showering and dressing, and medication management. While many patients received community support on a weekly or fortnightly basis, the highest level of care was three times a day.

The most frequent health issues on admission were shortness of breath related to exacerbations of Chronic Obstructive Pulmonary Disease (COPD) or asthma (*n* = 11), pain - including back or abdominal pain, headache, or pain related to the presence of kidney stones or cancer (*n* = 11), collapse (*n* = 6) and chest pain (*n* = 4). Several patients had more than one presenting problem.

Forty-four percent of patients remained in hospital for up to four days, and 30 percent for between five to eight days. A further quarter had lengthy inpatient stays; the longest admission was for 36 days. A majority of patients (72 %) had had a previous hospital admission during the past 12 months, and almost half (44 %) had been discharged from hospital within the previous two months. The sample also included two patients who had been re-admitted to hospital either on the same day or the day following discharge.

### Root Causes of Admissions

A case study illustrating the processes followed in the RCA analysis is presented in Additional file 1. The root causes of each admission was determined and subsequently categorised into different thematic groups. These categories and examples of specific root cause statements are outlined in Table 1. Six thematic categories were identified and are presented in order of the frequency (from highest to lowest) they were identified in the sample of admissions: minimal care, progression of disease, home care accessibility, high complexity clinical error and delayed care-seeking by the patient.

**Table 1:** Root cause thematic categories and examples

Category (N = 36)	Examples of root causes (number of cases)
Minimal care n = 8 (22.2 %)	Readmission after lack of diagnosis and treatment of symptoms causing multiple prior admissions (3)
	Poor integration of care between specialists and lack of outpatient follow-up (1)
	Insufficient treatment and lack of follow-up after diagnosis of major health problem (1)
	Lack of diagnosis and sufficient follow-up for major acute health condition (3)
Progression of disease n = 8 (22.2 %)	Failure of ambulatory treatment with oral antibiotics (1)
	Condition presenting with atypical symptoms until severe (1)
	Condition most appropriately managed in hospital, i.e. syncope (3), nose bleed not responding to usual measures (1)
	Progression of disease despite appropriate treatment (2)
Home care access n = 6 (16.7 %)	Homebound patient unable to access timely medical services in home environment (2)
	Homebound patient did not receive expected home visit from GP and unable to follow-up (1)
	Lack of access to community nursing and poor co-ordination of care (2)
	Patient did not want locum to come to home due to previous concern about late visit (1)
High complexity n = 5 (13.9 %)	Interaction between clinical care, patient behavior or characteristics, and inadequate social support (5)
Clinician error n = 5 (13.9 %)	Readmission after patient discharged before clinically stable (1)
	Readmission due to medication dose remaining unaltered after major change in health status (1)
	Readmission to hospital after failure to follow guidelines for treatment (1)
	Misdiagnosis over the telephone with subsequent treatment failure (1)
	Medication prescribing error (1)
Delayed care-seeking by patient n = 4 (11.1 %)	Patient with UTI on visit from country for several days refused to see daughter's GP (1)
	Patient did not want to bother GP for home visit while experiencing increasing shortness of breath from COPD (1)
	Delayed seeking of care for significant medical symptoms (2)

## Discussion

This study is the first application of root cause analysis to determine the reasons why older people with multiple morbidities are admitted to hospital. Using an innovative approach to examine the reasons why these admissions occurred, we were able to determine root causes for 36 hospitalisations to an acute medical unit. This study is unique in that it sought to ascertain the perspectives of all those involved in the patients’ care – including the patient themselves, family members, GPs, specialists and other healthcare providers. Previous research exploring the circumstances leading to hospital admissions of older adults, has either focused on patient perspectives alone, or when ascertaining GP opinion has used generic postal questionnaires rather than face-to-face interviews [20, 21]. The methodology used in the study provided rich detail to examine the reasons for admissions.

The root causes of the admissions to hospital were analysed and grouped into six different categories. These categories included minimal care, progression of disease, delayed care seeking by patients, medical error, home care accessibility, and high complexity.

Eight hospitalisations fit the primary category of minimal care which indicated circumstances where, while no clinician error was clearly identified, the care provided was focused on basic diagnostic testing or treatments. From this retrospective analysis of these hospitalisations, potentially important elements of care that might have been provided to prevent health deterioration and subsequent hospital admission did not occur. While in some circumstances appropriate medical investigations were initiated, the pace of evaluation was slower than the acute nature of the illness warranted. Failure to initiate timely follow-up was also a factor in several of these hospital admissions. This finding suggests that in both hospital and community settings sufficiently proactive care for this patient group was lacking.

In a further eight hospitalisations, the root cause of admission was identified as illness progression. In each instance, despite the actual health care provided being regarded as appropriate, the illness had either progressed or developed a new manifestation requiring acute medical care. Hence characteristics of the underlying illness were a key factor in determining need for hospital care; it was therefore considered that these admissions were not preventable.

In six hospitalisations, home care access issues occurred. In this situation, individuals who relied on home visits by GPs and nurses to meet their needs (usually due to mobility problems) were unable to obtain urgent access to services in their home. Home visits as a proportion of total Australian GP visits have been falling dramatically [22]. Although the at-risk patients in this study had access to routine home visits from their GP, the capacity to obtain urgent appointments was considerably limited. As Australian public hospitals continue to limit the provision of out-of-hospital services, regional primary care organisations (e.g. Primary Health Networks) are playing an increasingly important role supporting community health care. Special attention is required for this high-risk population to assist in the prevention of unnecessary inpatient admissions.

In five of the cases a specific root cause was difficult to determine and categorise as each admission occurred in the setting of a high degree of complexity. A range of issues was noted in the background of these patients which put them at high risk of hospitalisation, including mild cognitive impairment, substance abuse and low health literacy. However, in each case there was a clear interaction between an unstable clinical condition, patient characteristics or behavior, and the social context. The clinicians facing this web of complexity determined that referral to the emergency department, and subsequent admission to hospital, was the most expedient solution to address the patient’s health problems. However, a similar presentation of the condition in a different context might not have necessitated hospital admission. For these patients, the complexity of their health and social care needs exceeded the availability and responsiveness of the ambulatory health care system.

In the final four cases patients delayed seeking care despite experiencing significant symptoms. A number of reasons were provided for this delay, including not wanting to bother the doctor, inconvenience of seeking care, or not knowing who to call. A clear care plan, including an action plan to monitor for red flag conditions and how to respond to related health concerns, may have avoided delayed care-seeking. However, although care plans were present in a minority of medical records, none of the patients interviewed were aware of the existence of a care plan. More attention to the care planning process for the predictable consequences of chronic disease (e.g. exacerbation of COPD) and who to contact if increasing symptomatology occurs may assist in reducing hospital admissions. The Australian Department of Veterans Affairs Coordinated Veterans Care program requires a patient friendly care plan for veterans enrolled in this program which provides clear guidance regarding what to do if specific red flag symptoms develop including who to call and the urgency of getting a response. A similar process could be adopted within primary health care for other older adults with complex chronic disease.

### Limitations

Several limitations to this study should be acknowledged. This was an exploratory study and the purposive sampling method used means that the relative weighting given to each root cause category should not be over-interpreted. In addition the sampling frame did not include people who had significant cognitive impairment, did not speak English or who were too unwell to be interviewed. This means that the sample is not representative of all older people who experience unplanned hospital admissions. None-the-less the categories identified appeared to be robust with multiple root causes fitting into each thematic grouping.

A further limitation is that the RCA approach, although highly structured, is relatively new and as far as we are aware has not previously been used in this setting before. There are elements of investigator subjectivity in the manner in which different thematic categories are generated. However the team that performed this analysis (a general practitioner in active practice with a strong research interest in this area, a social worker with extensive clinical experience, and a hospital-based clinician responsible for clinical governance) were highly skilled in this methodology and familiar with the context. It would be valuable to confirm that the themes identified in this study are also present in other settings.

With respect to the possible broader dissemination of this approach it needs to be acknowledged that the multiple interviews required for each admission are very time consuming. The speed with which the team was able to review case data improved over the course of the study and it is possible that better efficiencies could be achieved by modifying and abbreviating some of the steps outlined in the present study.

## Conclusion

Despite these limitations, the study indicates that a range of factors other than disease characteristics impact on acute hospital admissions by older people with complex health issues. Addressing these causes, such as reducing medical errors, improving access to home visits for housebound older adults, and anticipating ‘red flag’ symptoms, may potentially decrease the rate of admissions. Use of the RCA methodology and classification approach outlined in the present study potentially could assist in developing interventions targeted to reducing hospitalisations in this high-risk population. A larger epidemiological cohort study is required to further explore the efficacy of this approach.

## Additional files

Additional file 1:
**Patient questions to guide semi-structured interviews.** (DOCX 110 kb) Additional file 2:
**Health professional questions to guide semi-structured interviews. **(DOCX 119 kb) Additional file 3:
**The following case study provides an example of the RCA processes followed in this study.** (DOCX 151 kb)
