# Supplementary material for: Why do older people with multi-morbidity experience unplanned hospital admissions from the community: a root cause analysis
Source: BMC Health Serv Res. 2015 Nov 27;15:525. doi: 10.1186/s12913-015-1170-z (PMC4662024; doi:10.1186/s12913-015-1170-z)
Supplement: Additional file 1: — Patient questions to guide semi-structured interviews. (DOCX 110 kb) [file 12913_2015_1170_MOESM1_ESM.docx]

Appendix 1

**Patient questions to guide semi-structured interviews**

Recent health

What was the reason why you had to come into hospital?

How long had you felt unwell for before coming into hospital?

What symptoms did you experience/any impact on mobility or ability to care for yourself?

Have there been any recent events in your life that you think may have contributed to your illness

Recent health - GP involvement

Did you see your GP about the problem?

When you saw your GP, how long had you been feeling unwell?

Were you able to get an appointment with your GP as soon as you needed it?

Were you able to see the GP of your choice?

Were you satisfied with the consultation/your relationship with your GP?

Did you understand/were happy/feel involved with the treatment plan?

Did someone (family member, carer) attend the consultation with you?

If yes, were they involved in discussions regarding the treatment of your illness?

How often have you seen your GP since you first felt unwell?

When was the last time that you saw your GP?

Recent health – other health providers

Were you referred to any specialists or other health providers? If yes, who did you see?

Did you have to wait to get an appointment? Was the length of waiting time acceptable to you?

Were you happy with the consult/treatment plan/ care?

General health

Do you have any physical health problems (other than the problem that you came to hospital for?

Do you have any mental health problems or difficulties with your memory?

Are yon receiving any treatment or medication for these problems?

How many types of medications do you take (prescription/non-prescription)?

Do you have any problems with the medication (remembering to take it at the required time, getting the medication, adverse effects, only take it when you feel you need it)?

How do you rate your mobility?

Home Situation

What is your living situation (type of residence; lives alone/with others)?

Do you have family and friends who you are in contact with or get support from?

Do you receive any support at home (health care providers, home care, home alert, community transport, equipment or aids)?

Have you ever been offered support at home but have declined the offer? Why?

How well do you feel that you cope at home? Do you have any difficulties with sleep, falls, continence, cooking (including nutrition), cleaning, and personal care?

Arc you happy with the current level of support that you receive? If not, why?

Recent health- summary

Overall, how do you feel about the care that you have received for your illness?

Do you think that this episode of illness/hospital admission could have been avoided? If yes, how?

Do you have any other comments that you would like to make or questions that you would like to ask?
