# Supplementary material for: Why do older people with multi-morbidity experience unplanned hospital admissions from the community: a root cause analysis
Source: BMC Health Serv Res. 2015 Nov 27;15:525. doi: 10.1186/s12913-015-1170-z (PMC4662024; doi:10.1186/s12913-015-1170-z)
Supplement: Additional file 2: — Health professional questions to guide semi-structured interviews. (DOCX 119 kb) [file 12913_2015_1170_MOESM2_ESM.docx]

**Appendix 2. Health professional questions to guide semi-structured interviews**

(note these were tailored to individual patient circumstances – fictional example used)

Mr Jones was discharged from the ABC hospital on 1 April and admitted to Flinders Medical Centre on 14th April. Did you receive any communication from the hospital regarding the admission (e.g. telephone calls, discharge summary)? Was the communication sufficient to meet your needs?

What is your opinion of the care Mr Smith received in hospital? Were the investigations and treatments appropriate? Have the causes for admission been resolved satisfactorily or are there issues left unaddressed?

Mr Jones was treated in hospital for heart failure. How often did you see/have contact with Mr Smith since his discharge from ABC hospital? When did you last see Mr Jones? What were your impressions of his health/were you aware that his health had deteriorated? What was your treatment?

Does Mr Jones have a history of heart failure? What is his usual heart function? Has he had regular testing?

How is his condition managed? Has he seen a specialist in the past for this issue? Were you aware that Mr Jones was being admitted for surgery? Did you have any concerns regarding the doses of his medications post-surgery?

Mr Jones reported that he has had liver problems in the recent past. What is his history of liver disease?

What specialist input has he been receiving? Do you receive communications from these services?

Mr Jones reported that he had contact with an out-of-hours GP service prior to his admission to hospital. What are the out-of-hours arrangements for your practice?

What is your opinion of the service? Did you receive any communication from them regarding Mr Jones attendance? Is this usual?

Are there any changes that you would like to see put in place which would make it easier for you as a GP working with older patients like Mr Jones but also in general?
