# Supplementary material for: Why do older people with multi-morbidity experience unplanned hospital admissions from the community: a root cause analysis
Source: BMC Health Serv Res. 2015 Nov 27;15:525. doi: 10.1186/s12913-015-1170-z (PMC4662024; doi:10.1186/s12913-015-1170-z)
Supplement: Additional file 3: — The following case study provides an example of the RCA processes followed in this study. (DOCX 151 kb) [file 12913_2015_1170_MOESM3_ESM.docx]

**Appendix 3**

The following case study provides an example of the RCA processes followed in this study.

A 78 year old male was admitted to the Acute Assessment Unit with constipation, shortness of breath, reduced appetite, fever and chills. The patient had been referred to the Emergency Department by his GP following an appointment on the day of admission – the GP suspected that the patient may be suffering from cholangitis. With a history of COPD (the patient used home oxygen) and cardiac failure, the patient had had 31 previous admissions, primarily for issues relating to his COPD. The patient had regular contact with the Respiratory nurses at hospital but was not currently being monitored by the Respiratory Clinic (the patient had not attended an appointment and whilst a note in the file stated “next available appointment” a further appointment was not arranged). During his contacts with the respiratory nurses, the patient had been given information regarding additional services for people with COPD. However, the patient discharged himself after an initial assessment with the Chronic Disease Management Program, and did not wish to attend Pulmonary Rehabilitation or a lung support group.

The patient was interviewed during his admission to hospital and his medical notes were reviewed. Subsequent interviews were conducted with the patient’s wife, GP, and respiratory nurses.

Event flow diagram:

**Week 1**

- Diarrhoea then constipation – taking regular fruit laxative

**Week 3**

- Worsening constipation
- GP appointment – medication for

urinary problems

**Week 7**

- Worsening constipation
- GP appointment – scripts/blood tests
- Developed bronchitis – started antibiotics

**Week 8 (Day of admission)**

- Felt “really crook”
- Not slept, hard stomach, not fully passed faeces/ urine
- SOB – concerned may be developing

pneumonia

**Day of admission**

- GP appointment – sent to ED with referral letter
- ED - admitted to AAU

**Why?**

- Is this an ongoing problem?
- Has this been discussed with GP/education been provided?
- Is there a plan for addressing constipation?

**Why?**

- Was GP informed of constipation/

provide advice?

- Why was the medication prescribed – to treat urinary frequency or retention?
- What follow-up was arranged?
- How often does the patient see the GP?

**Why?**

- Was GP informed of constipation/provide advice?
- What was the purpose of the blood test?
- How are antibiotics obtained - emergency pack?
- What is the plan for management of COPD/who has responsibility for plan?
- Does patient follow management plan?

**Why?**

- Any contact with respiratory clinic?
- What communication does GP receive from specialist services/

opinion of communication and services received?

- Are there protocols for treatment of COPD – is GP aware of these?

**Why?**

- What were the contents of the referral letter/GP’s

main concerns?

- What information did the GP receive from the patient?

Cause and effect diagram:

Patient admitted to hospital

Suspected cholangitis – abdominal pains, abnormal LFTs, fever, chills, SOB

No alternative method for rapid assessment available

Review of rapid assessment systems has not been undertaken

Constipation (worsening over 8/52)

Patient attempted to manage own condition & did not discuss with GP

Unstable COPD (mimicking other conditions)

COPD not optimally treated due to lack of comprehensive management plan

History of self-management with fruit laxative

Lifestyle factors – poor diet and lack of exercise

No communication between respiratory nurses and GP

Non-participation in self-management activities

Local COPD protocols not disseminated to GP

Not monitored by Respiratory Clinic – follow-up not arranged after missed appointment

Patient & wife not following plan - unaware of management plan

**ROOT CAUSE**

**ROOT CAUSE**

**ROOT CAUSE**

Root cause statements:

1. As there was no other method for rapid assessment of the patient’s suspected cholangitis available to the GP, this increased the probability that the GP would have to refer the patient to acute services.
2. Due to the patient attempting to self-manage his constipation and not discussing this problem with his GP, a plan to address the constipation was not implemented. This led to an acute episode of constipation on the day of admission.
3. A lack of a comprehensive management plan for the patient’s COPD contributed to his unstable condition. This increased the likelihood of episodes of shortness of breath requiring acute care assessment.

Recommendations:

1. A review of options for rapid patient assessment for patients with chronic diseases to be undertaken.
2. A management plan for constipation is devised by the GP and followed by the patient and GP in order to prevent or address future episodes of constipation. This plan should include education for the patient regarding lifestyle factors such as diet and exercise.
3. An updated written management plan for COPD is devised by the GP in collaboration with the patient (with guidance from the respiratory clinic and respiratory nurses) and followed by the patient and GP.
4. Local COPD guidelines to be disseminated to all GP practices.
